# Author Correction: Evolution from unimolecular to colloidal-quantum-dot-like character in chlorine or zinc incorporated InP magic size clusters

**DOI:** 10.1038/s41467-021-22007-4

**Published:** 2021-03-08

**Authors:** Yongju Kwon, Juwon Oh, Eunjae Lee, Sang Hyeon Lee, Anastasia Agnes, Gyuhyun Bang, Jeongmin Kim, Dongho Kim, Sungjee Kim

**Affiliations:** 1 Department of Chemistry, Pohang University of Science and Technology, Pohang, 37673 South Korea; 2 Department of Chemistry and Spectroscopy Laboratory for Functional π-Electronic Systems, Yonsei University, Seoul, 03722 South Korea; 3 Department of Chemistry, University of California, Berkeley, CA 94720 USA

**Keywords:** Nanoparticles, Quantum dots, Synthesis and processing

Correction to: *Nature Communications* 10.1038/s41467-020-16855-9, published online 19 June 2020.

The original version of this Article omitted the following from the Acknowledgements:

This work was also supported by SAIT, Samsung Electronics Co., Ltd.

This has now been corrected in both the PDF and HTML versions of the Article.

