# Supplementary material for: Evolution from unimolecular to colloidal-quantum-dot-like character in chlorine or zinc incorporated InP magic size clusters
Source: Nat Commun. 2020 Jun 19;11:3127. doi: 10.1038/s41467-020-16855-9 (PMC7305325; doi:10.1038/s41467-020-16855-9)
Supplement: Supplementary file 1 — Supplementary Information [file 41467_2020_16855_MOESM1_ESM.pdf]

Supplementary Information

Evolution from Unimolecular to Colloidal-Quantum-Dot-like Character in Chlorine or Zinc Incorporated InP Magic Size Clusters

Yongju Kwon†, Juwon Oh‡,§, Eunjae Lee†, Sang Hyeon Lee‡, Anastasia Agnes†, Gyuhyun Bang†, Jeongmin Kim†, Dongho Kim*‡ and Sungjee Kim*†

Y. Kwon and J. Oh contributed equally to this work.

† Department of Chemistry, Pohang University of Science and Technology, Pohang 37673, South Korea

‡ Department of Chemistry and Spectroscopy Laboratory for Functional π-Electronic Systems, Yonsei University, Seoul 03722, South Korea

§ Department of Chemistry, University of California, Berkeley, California 94720, United States

Supplementary Methods

Time-Resolved PL Measurement. A time-correlated single photon-counting (TCSPC) system was used to measure the spontaneous photoluminescence decay. The excitation light source was a mode-locked Ti:sapphire laser (Spectra-Physics, MaiTai BB) which provides ultrashort pulse (center wavelength 710~990 nm with 80 fs at FWHM with high repetition rate (80 MHz). This high repetition rate was reduced to 200 kHz by using a homemade pulse picker. The frequency of the pulse-picked output was doubled by a 1-mm thick BBO crystal (type I, $\theta = 29.2°$, EKSMA). The photoluminescence was collected by a microchannel plate photomultiplier (MCP-PMT, Hamamatsu, R3809U-51) with a thermoelectric cooler (Hamamatsu, C10373) connected to a TCSPC board (Becker; Hickel SPC-130). The overall instrumental response function was ~ 25 ps FWHM. A pump pulse that had been vertically polarized by a Glan-laser polarizer was shone on samples, and a sheet polarizer set at 35.3° (i.e., complementary to the magic angle) was placed in the photoluminescence collection path to obtain polarization-independent photoluminescence decays.

Direct synthesis of 386-OA InP MSCs. 386-OA InP MSCs were synthesized in almost the same way as 386-MA InP MSCs.[1] First, 0.8 mmol of $In(Ac)_3$ and 2.9 mmol of HOA in a three-necked round-bottom flask containing 20 ml of ODE was degassed for 2 h at 110°C then 0.4 mmol of $(TMS)_3P$ was added to the mixture and it was stirred for 2 h under N_2 atmosphere.

Direct synthesis of OA capped F360-InP:Zn MSCs. First, 0.6 mmol of $In(Ac)_3$ and 1.2 mmol of ODPA were prepared in 12 ml ODE. The solution was degassed for 2 h at 110°C and heated for 2 h at 300°C, then degassed again for 2 h at 110°C. in another pot, a solution of 0.6 mmol of zinc oxide and 1.5 mmol of HOA in 10 ml ODE was degassed for 1 h at 110°C, then heated to 300°C for 2 h, then transferred into the indium phosphonate solution at RT. Then 0.3 mmol of $(TMS)_3P$ in 2 ml ODE was injected into the mixture solution at RT and the solution was heated to 180°C. Aliquots were taken during synthesis to confirm the end of the reaction (~28 h).

Direct synthesis of OA capped F408-InP:Zn MSCs. OA-capped F408-InP:Zn MSCs were synthesized from molecular precursors in almost the same way as OA-capped F360-InP:Zn MSCs. First, 0.6 mmol of $In(Ac)_3$ and 0.9 mmol of ODPA were prepared in 12 ml ODE. The solution was degassed for 2 h at 110°C and heated for 2 h at 300°C then degassed again for 2 h at 110°C. In another pot, a solution of 0.6 mmol of zinc oxide and 1.5 mmol of HOA in 10 ml ODE was degassed for 1 h at 110°C and heated to 300°C for 2 h, then transferred into the indium phosphonate solution at RT. Then 0.3 mmol of $(TMS)_3P$ in 2 ml ODE was injected into the mixture at RT and the solution was heated to

300°C Aliquots were taken during synthesis to confirm the end of the reaction. After ~3 h, the absorption peak at 408 nm emerged.

Direct synthesis of OA capped F393-InP:Zn MSCs. OA-capped F393-InP:Zn MSCs were synthesized from molecular precursors in almost the same way as F360-InP:Zn MSCs. First, 0.6 mmol of $In(Ac)_3$ and 0.11 mmol of ODPA were prepared in 12 ml ODE. The solution was degassed for 2 h at 110°C and heated for 2 h at 300°C then degassed again for 2 h at 110°C. In another pot, a solution of 0.6 mmol of zinc oxide and 1.5 mmol of HOA in 10 ml ODE was degassed for 1 h at 110°C and heated to 300°C for 2 h, then transferred into indium phosphonate solution at RT. Then 0.3 mmol of $(TMS)_3P$ in 2 ml ODE was injected into the mixture at RT and the solution was heated to 300°C. Aliquots were taken during synthesis to confirm the end of the reaction. After ~10 h, the absorption peak at 393 nm emerged.

Supplementary Figures

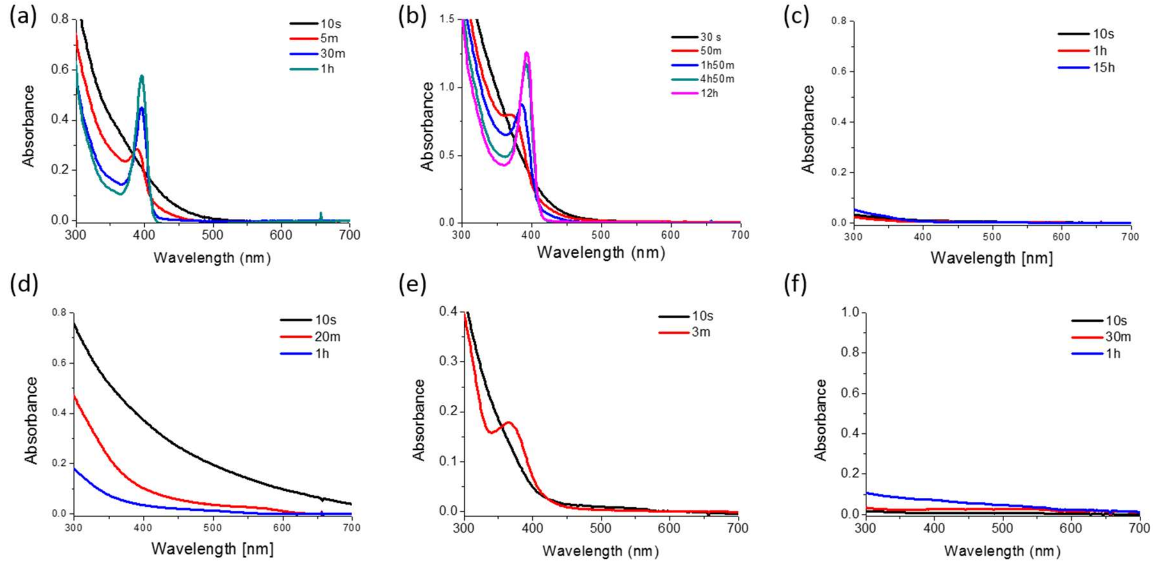

Supplementary Figure 1. UV−vis absorption spectra of aliquots taken during direct synthesis of InP:Cl MSCs from $In(Ac)_3$, HMA, $InCl_3$, and $(TMS)_3P$ at (a) 110°C, (b) 80°C, (c) 25°C and (d) 150°C. The molar ratio of $In(Ac)_3$: HMA : $InCl_3$: $(TMS)_3P$ is 1:3:0.6:0.5. UV−vis absorption spectra of aliquots taken during direct synthesis of InP:Cl MSCs from $In(Ac)_3$, HMA, $InCl_3$, and $(TMS)_3P$ with molar ratios (e) 1:3:0.08:0.5 and (f) 1:3:1:0.5 at 80°C.

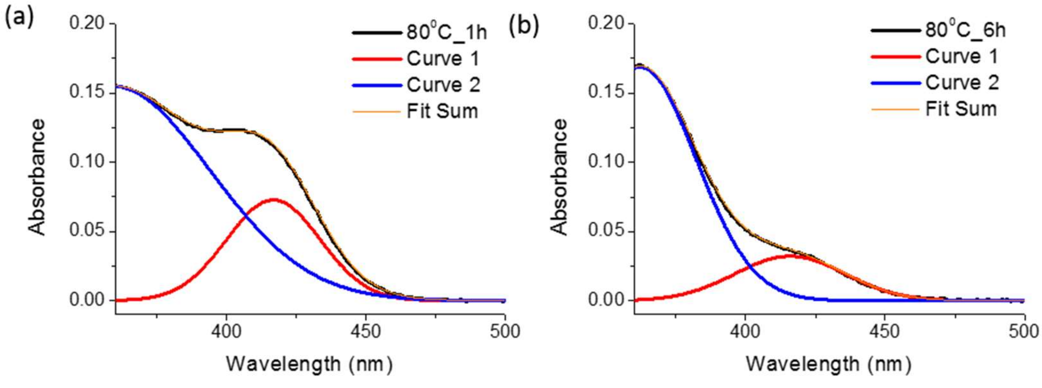

Supplementary Figure 2. UV-vis absorption spectra showing fitting of the mixture clusters, F360-InP:Cl MSCs and 416 nm intermediates (416-IS), during conversion synthesis of F360-InP:Cl MSCs from as-synthesized 386-InP MSCs using 56 equivalents of $InCl_3$ at 80°C. Conversion reaction time is (a) 1 h and (b) 6 h. Fitted peaks are represented by red (curve 1) and blue (curve 2) lines, and the sum of the fitted functions is represented by yellow line. The wavelength of maximum absorbance of the blue line was fixed at 360 nm while the remaining parameters were fitted.

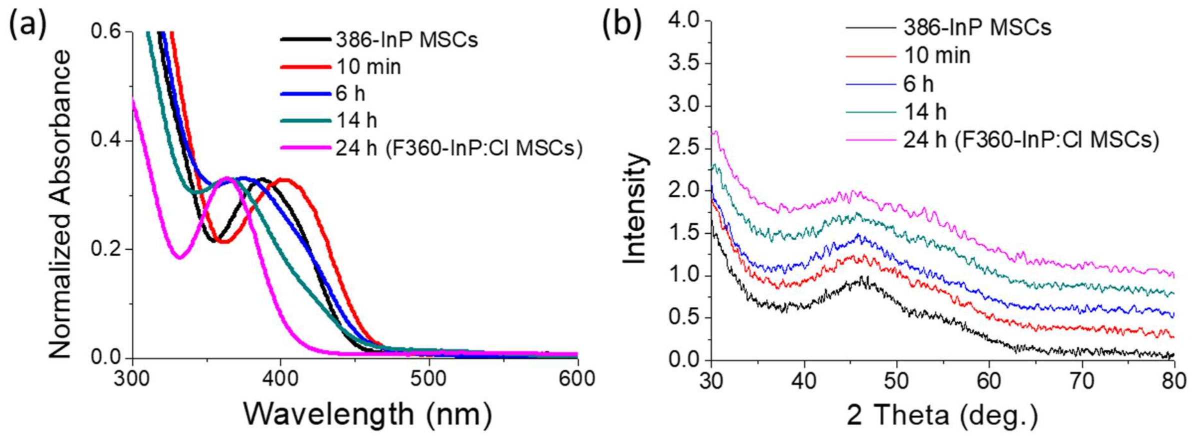

(c)

	386-InP MSC	10 min	6 h	14 h	F360-InP:Cl MSC
In	1.00	1.00	1.00	1.00	1.00
P	0.54	0.39	0.40	0.35	0.32
Cl	-	0.23	0.28	0.27	0.38

Supplementary Figure 3. (a) UV−vis absorption spectra, (b) XRD patterns and (c) EDX data of aliquots taken during conversion of F360-InP:Cl MSCs from 386-InP MSCs using as-synthesized 386-InP MSCs and 56 equivalents of $InCl_3$ at room temperature.

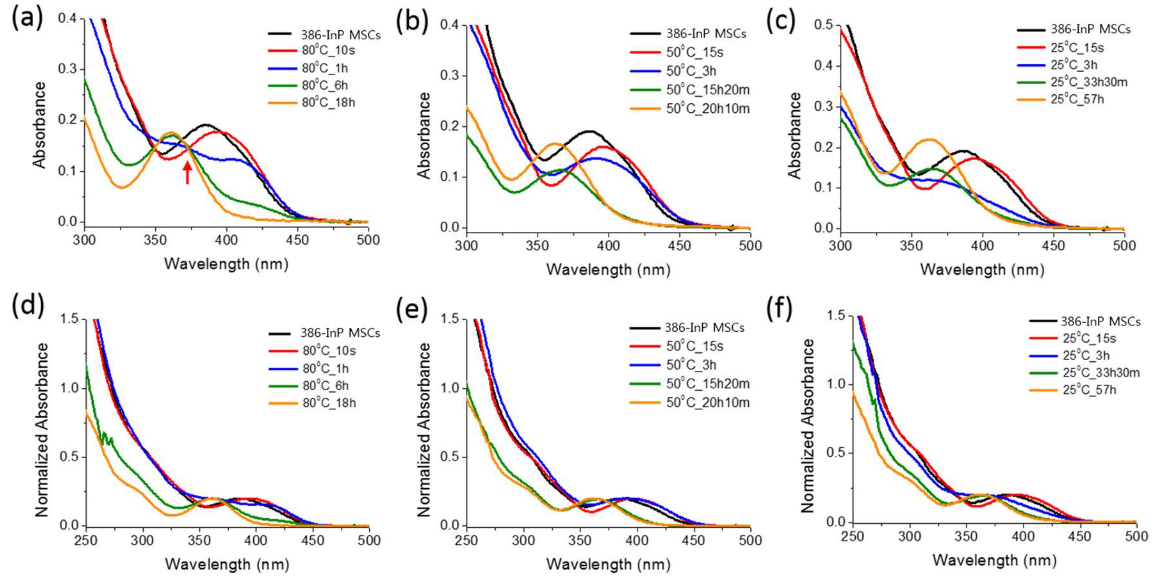

Supplementary Figure 4. UV−vis absorption spectra of aliquots taken during conversion synthesis of F360-InP:Cl MSCs from as-synthesized 386-MA InP MSCs using 56 equivalents of $InCl_3$ at (a) 80°C, (b) 50°C, and (c) 25°C. To show UV absorption change, absorption spectra of (a), (b), and (c) were normalized to the dominant absorption peak in (d), (e), and (f) respectively.

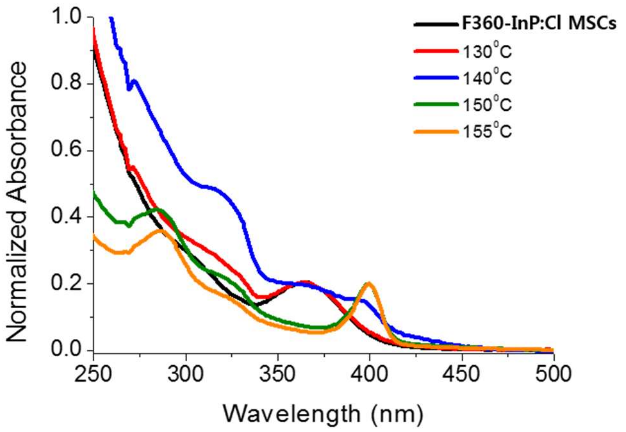

Supplementary Figure 5. Normalized UV−vis absorption spectra of aliquots taken during conversion synthesis of F399-InP:Cl MSCs from as-synthesized F360-InP:Cl MSCs by heating. Normalization was done by comparison to LEET.

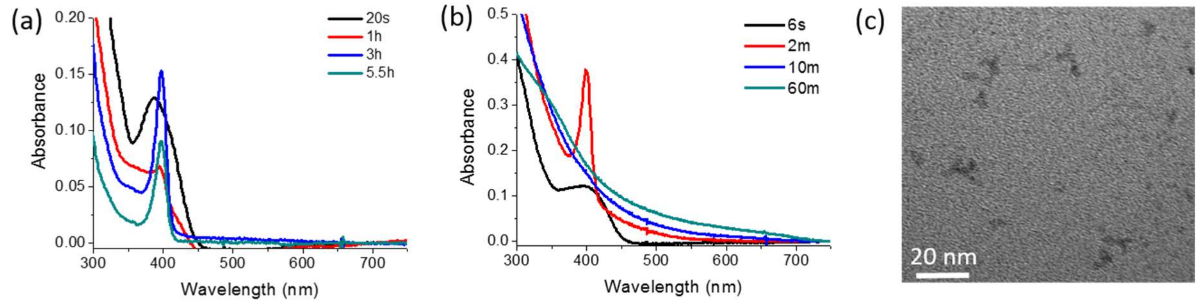

Supplementary Figure 6. UV−vis absorption spectra of aliquots taken during conversion synthesis of F399-InP:Cl MSCs from as-synthesized 386-MA InP MSCs using 56 equivalents of $InCl_3$ at (a) 110°C and (b) 180°C. (c) TEM image of grown InP nanoparticles after conversion of F399-InP:Cl MSCs from 386-InP MSCs.

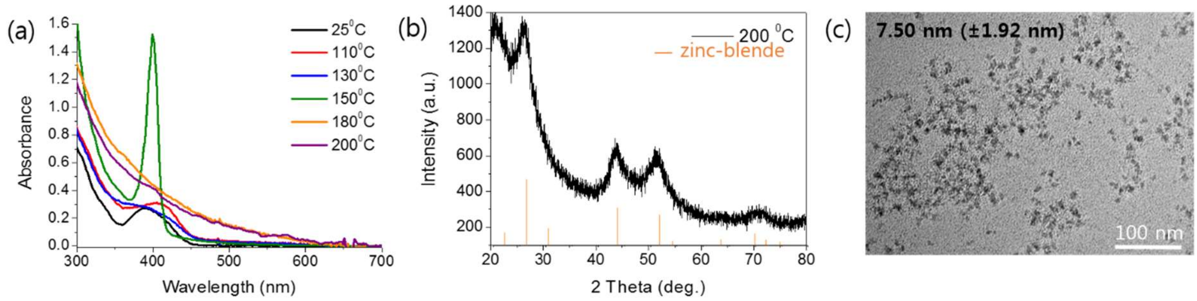

Supplementary Figure 7. (a) UV−vis absorption spectra of aliquots taken over time during transformation of QDs from F399-InP:Cl MSCs using as-synthesized 386-MA InP MSCs and 56 equivalents of InCl3, (b) XRD pattern and (c) TEM image of InP QDs transformed from F399-InP:Cl MSCs.

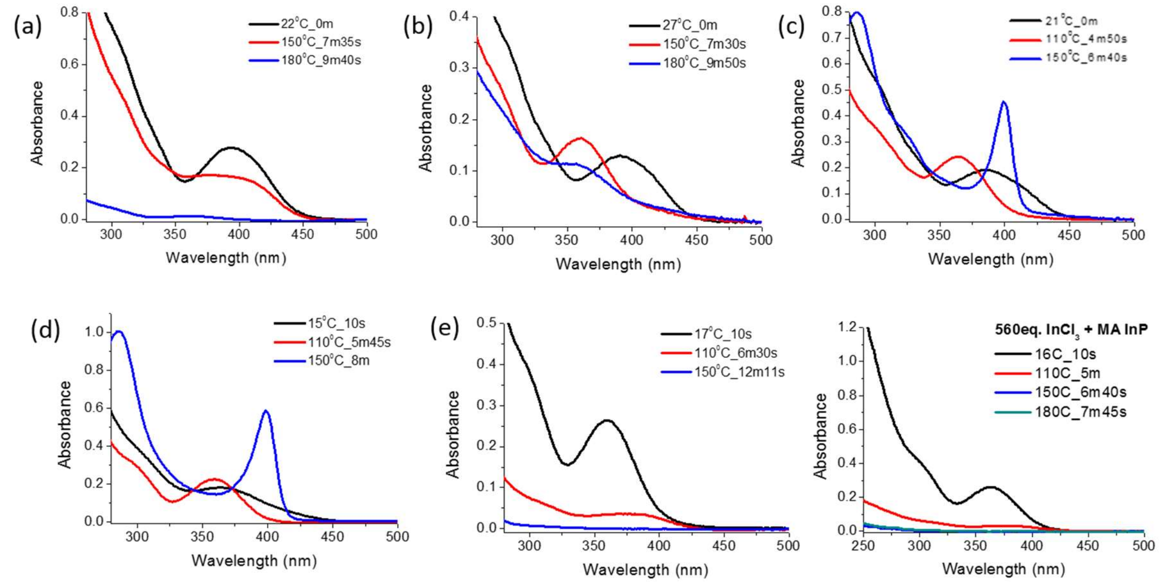

Supplementary Figure 8. UV−vis absorption spectra of aliquots taken during conversion synthesis of InP:Cl MSCs from as-synthesized 386-MA InP MSCs using (a) 28, (b) 40, (c) 56, (d) 112, (e) 280, and (f) 560 equivalents of $InCl_3$ by heating.

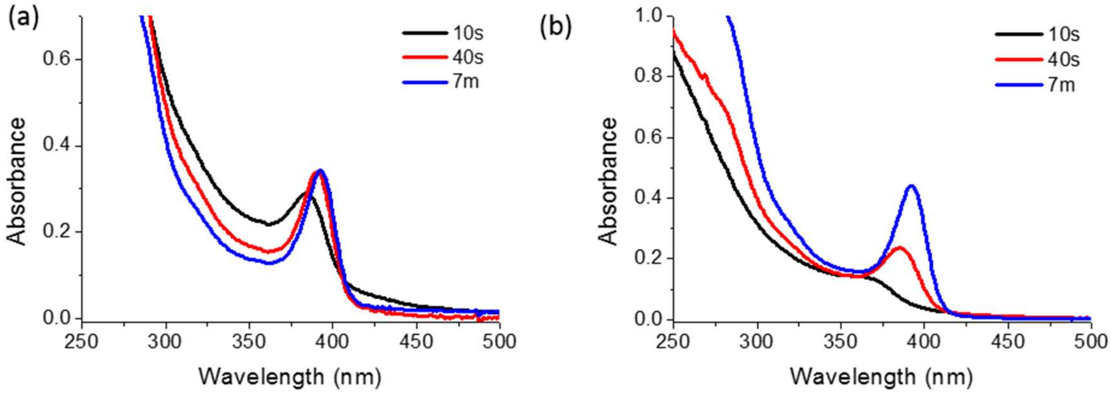

Supplementary Figure 9. UV−vis absorption spectra of aliquots taken during conversion synthesis of F399-InP:Cl MSCs from as-synthesized 386-MA InP MSCs using (a) 280 and (b) 560 equivalents of $InCl_3$ at 110°C.

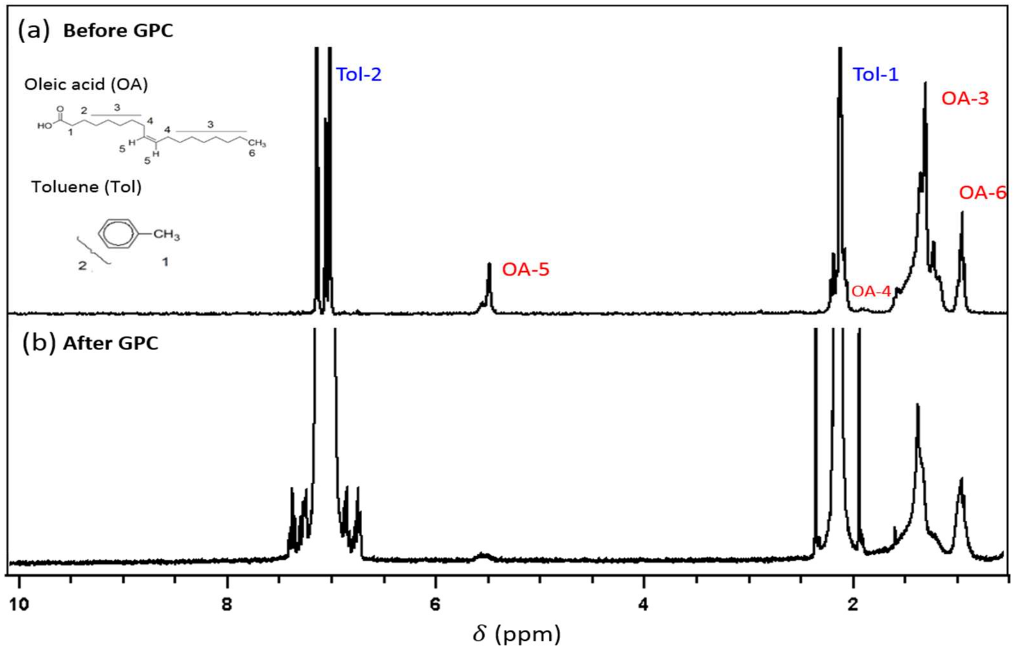

Supplementary Figure 10. ^{1}H NMR spectra of (a) as-synthesized 386-OA InP MSCs and (b) purified 386-OA InP MSCs by GPC method. Samples were first dispersed in toluene, then mixed with toluene-d8 for NMR measurement.

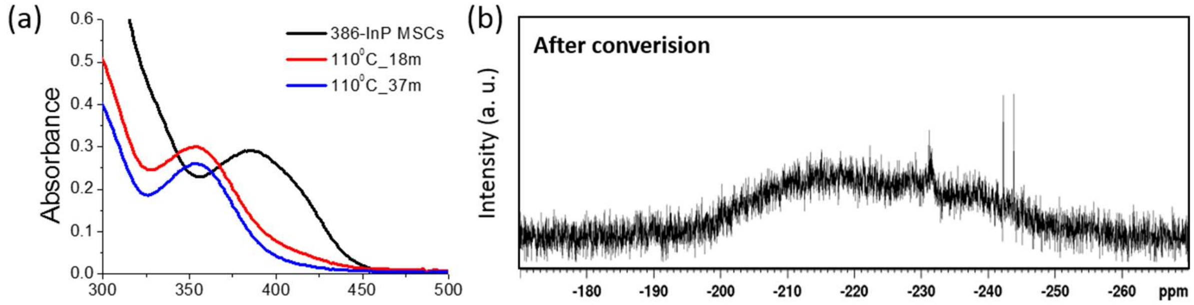

Supplementary Figure 11. (a) UV-vis absorption spectra and (b) $^{31}P\{^{1}H\}$ NMR spectrum of converted F360-InP:Cl MSCs from 386-OA InP MSCs using 25 % aqueous hydrochloric acid solution. Aqueous hydrochloric acid solution was injected into 386-OA InP MSCs solution at 110°C.

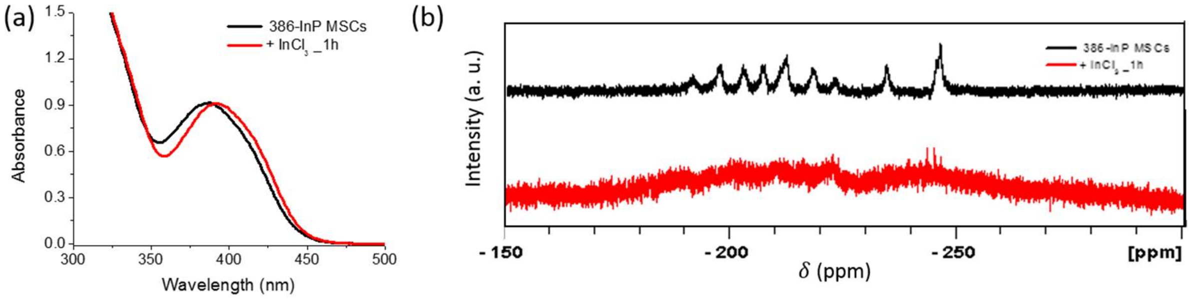

Supplementary Figure 12. (a) UV-vis absorption spectra and (b) $^{31}P\{^{1}H\}$ NMR spectrum of 386-OA InP MSCs and intermediates having 393 nm of dominant absorption peak formed as a result from the reaction of as-synthesized 386-OA InP MSCs with 90 equivalents of $InCl_3$ at room temperature for 1 h. The absorption peak red-shifted to 393 nm; this change is indicative of co-existence of 386-InP MSCs and 416-IS. The NMR spectra clearly showed the transition from 10 well-resolved peaks to broadened peaks with an extra distinct quartet peak at -243 ppm, which is assigned to PH_3.

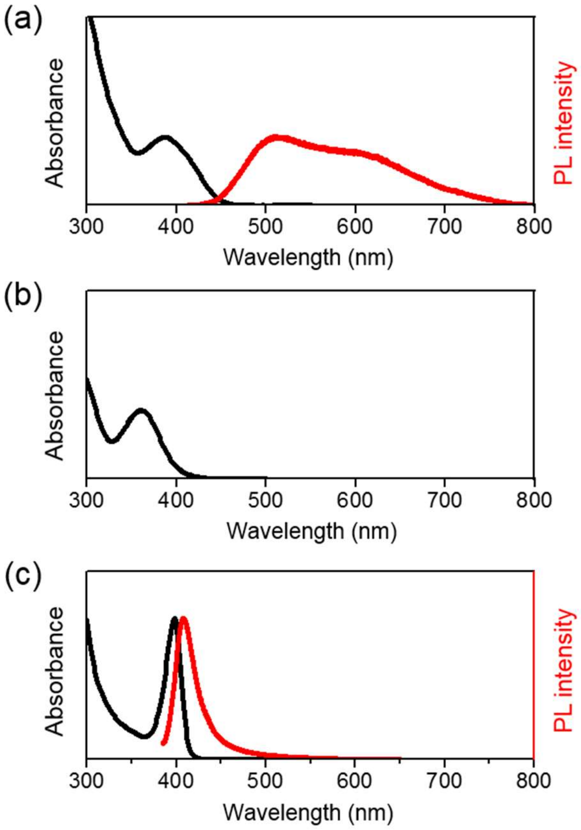

Supplementary Figure 13. UV−vis absorption and PL spectra of (a) 386-InP MSCs, (b) F360-InP:Cl MSCs, and (c) F399-InP:Cl MSCs. PL spectra of 386-InP MSCs and F399-InP:Cl MSCs were measured by excitation at 350 nm. F360-InP:Cl MSCs was non-emissive.

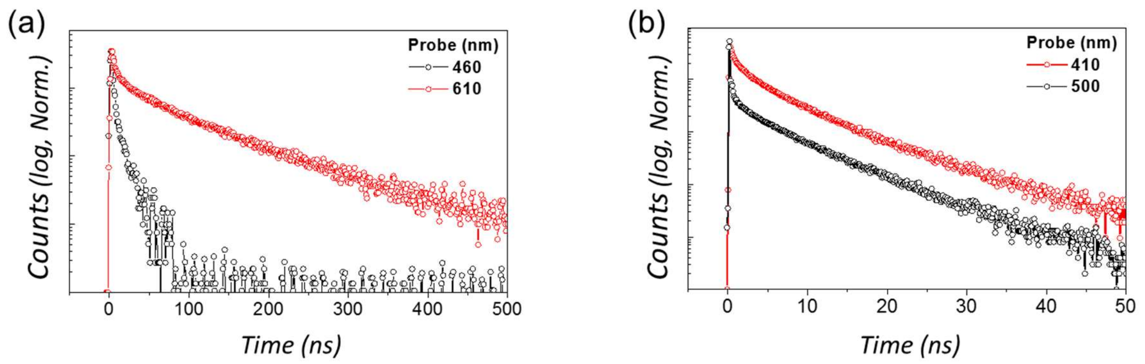

Supplementary Figure 14. Time-resolved PL decay profile of (a) 386-InP MSCs and (b) F399-InP:Cl MSCs.

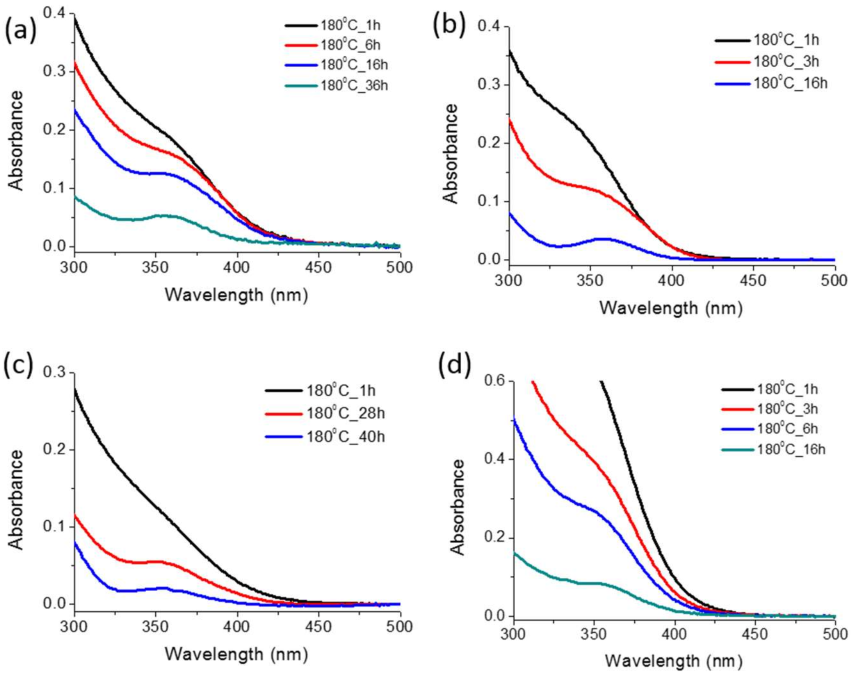

Supplementary Figure 15. UV−vis absorption spectra of aliquots taken during direct synthesis of F360-InP:Zn MSCs from $In(Ac)_3$, ODPA, $(TMS)_3P$, and $Zn(SA)_2$ precursors at 180°C. Molar ratio of $In(Ac)_3$:$Zn(SA)_2$:$(TMS)_3P$ is (a) 1:1.2:0.5, (b) 1:0.8:0.5, (c) 1.2:1:0.5, and (d) 0.8:1:0.5. The amount of ODPA was fixed to twice the amount of $In(Ac)_3$.

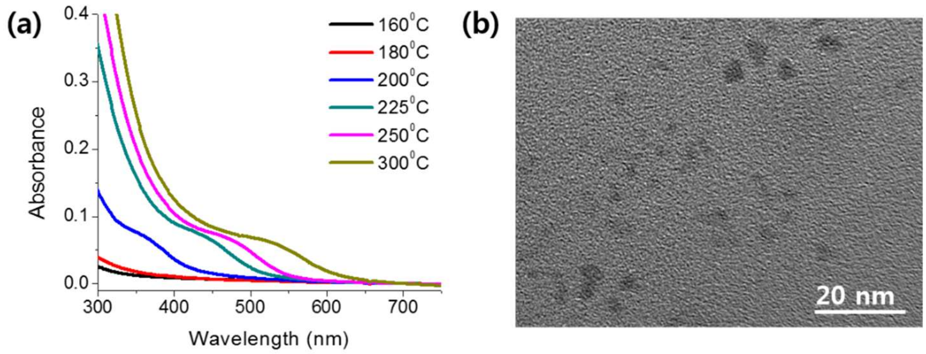

Supplementary Figure 16. (a) UV−vis absorption spectra of aliquots taken over time upon heating up to 300°C and (b) TEM images of the final samples for the reaction of $In(Ac)_3$, $Zn(SA)_2$, and $(TMS)_3P$ without ODPA.

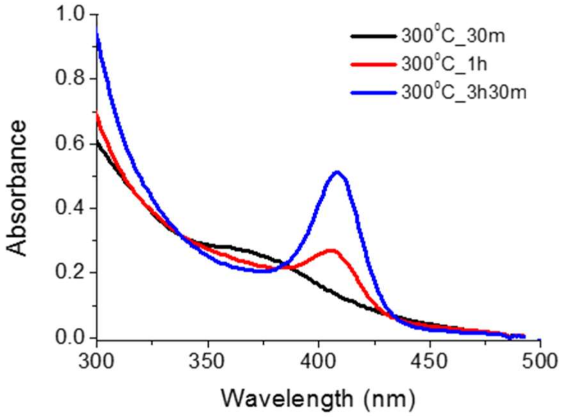

Supplementary Figure 17. UV−vis absorption spectra of aliquots taken during direct synthesis of F408-InP:Zn MSCs from $In(Ac)_3$, ODPA, $(TMS)_3P$, and $Zn(SA)_2$ precursors at 300°C. The molar ratio of $In(Ac)_3$: ODPA : $Zn(SA)_2$ is 1:1.5:1.

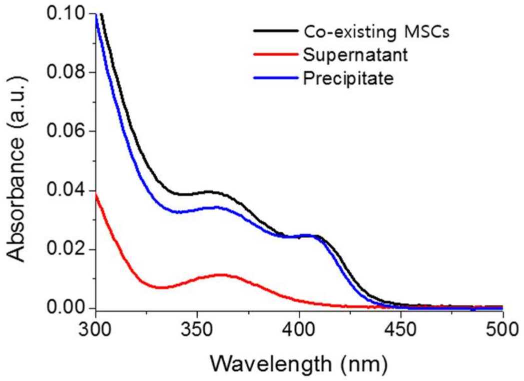

Supplementary Figure 18. UV-vis absorption spectra of early stage aliquot (black) during conversion of F408-InP:Zn MSCs from F360-InP:Zn MSCs; supernatant (red) and precipitate (blue) of the aliquots after ultracentrifugation.

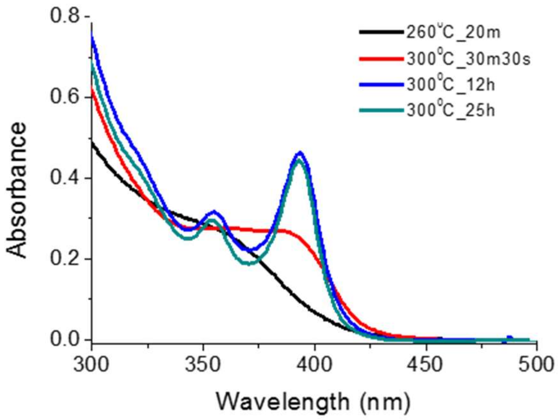

Supplementary Figure 19. UV−vis absorption spectra of aliquots taken during direct synthesis of F393-InP:Zn MSCs from $In(Ac)_3$, ODPA, $(TMS)_3P$, and $Zn(SA)_2$ precursors at 300°C. The molar ratio of $In(Ac)_3$: ODPA : $Zn(SA)_2$ is 1:1.85:1.

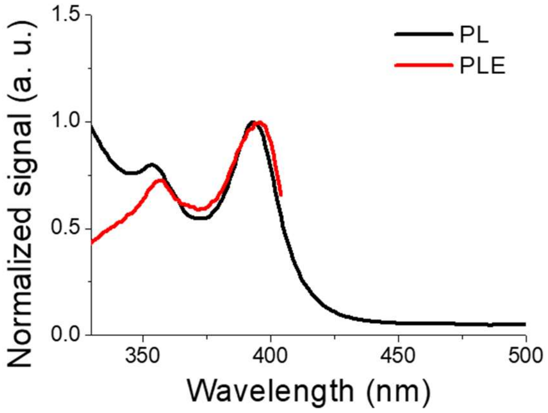

Supplementary Figure 20. UV-vis absorption and PLE spectrum of F393-InP:Zn MSCs. Excitation spectra collected for the band edge emission energies with 410 nm.

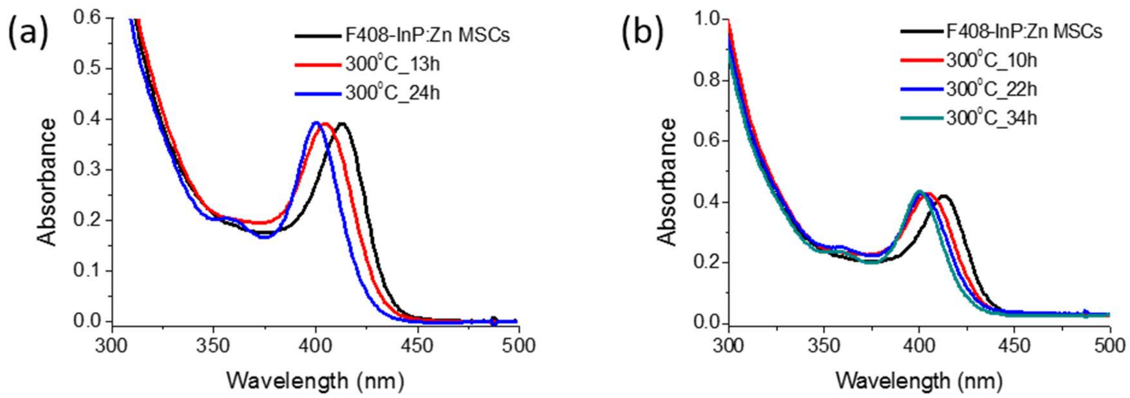

Supplementary Figure 21. UV−vis absorption spectra of aliquots taken during conversion synthesis of F393-InP:Zn MSCs (a) from direct-synthesized F408-InP:Zn MSCs and (b) from conversion-synthesized F408-InP:Zn MSCs by further heating at 300°C.

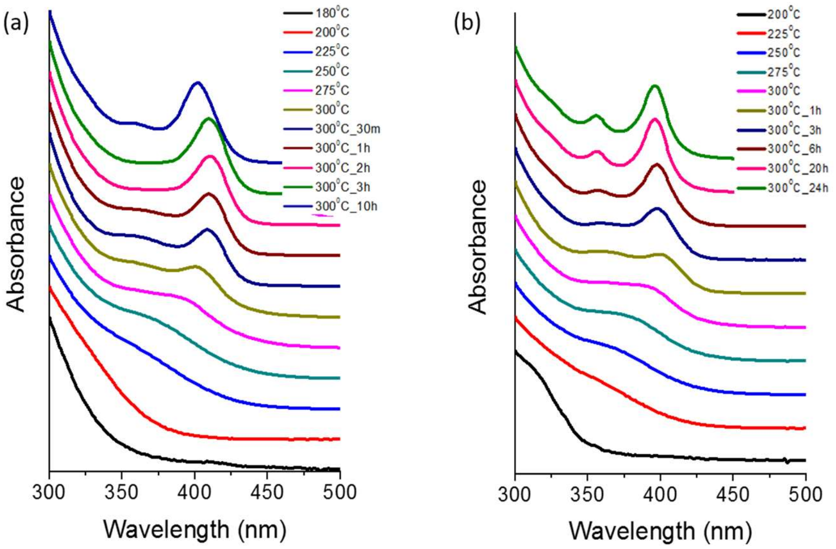

Supplementary Figure 22. UV−vis absorption spectra of aliquots taken during direct synthesis of F393-InP:Zn MSCs from In(Ac)$_3$, ODPA, (TMS)$_3$P, and Zn(SA)$_2$ precursors. The molar ratio of In(Ac)$_3$, ODPA, Zn(SA)$_2$, to (TMS)$_3$P is (a) 1:1.5:1:0.5 and (b) 1:1.85:1:0.5. Curves have been offset for clarity.

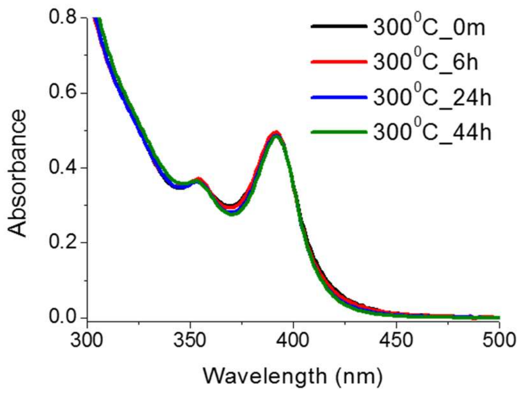

Supplementary Figure 23. UV−vis absorption spectra of aliquots taken during heating F393-InP:Zn MSCs solution at 300°C.

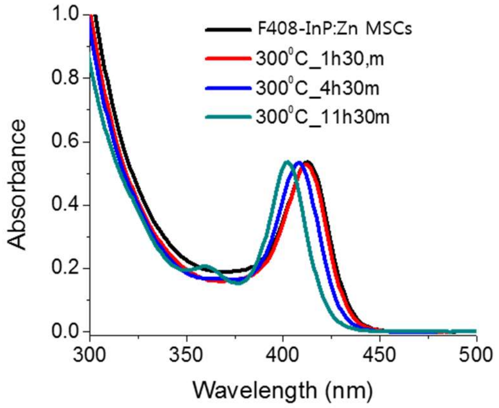

Supplementary Figure 24. (a) UV-vis absorption spectra of aliquots taken during conversion synthesis of F393-InP:Zn MSCs from F408-InP:Zn MSCs. The F408-InP:Zn MSCs was synthesized using zinc oleate instead of $Zn(SA)_2$.

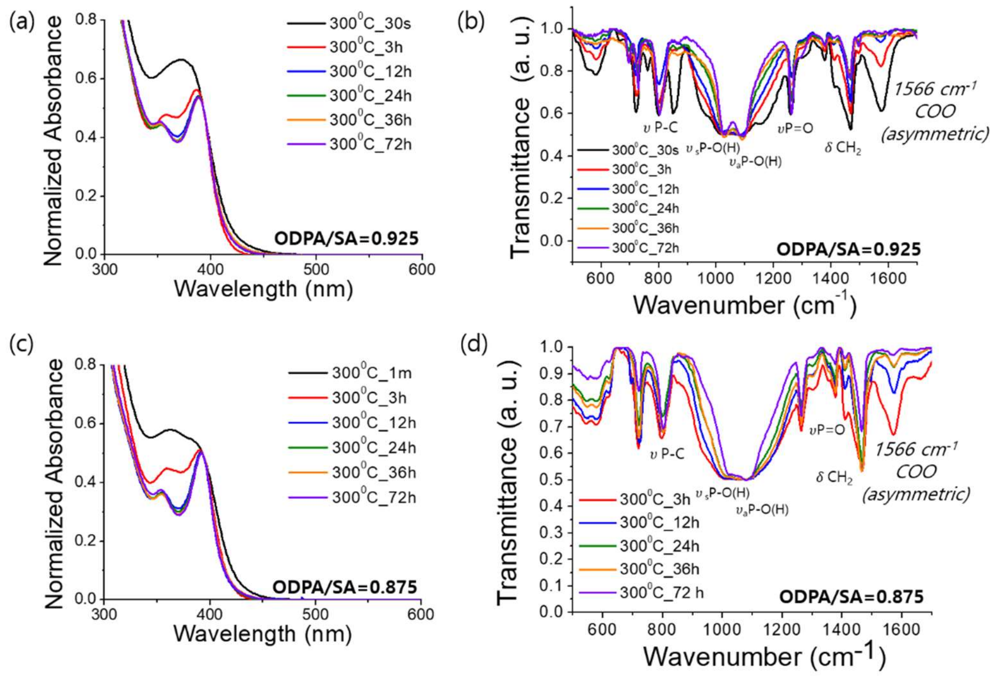

Supplementary Figure 25. (a) UV−vis absorption spectra and (b) FT-IR spectra of aliquots taken during direct synthesis of F393-InP:Zn MSCs from $In(Ac)_3$, ODPA, $(TMS)_3P$ and $Zn(SA)_2$ precursors at 300°C. The molar ratio of $In(Ac)_3$ to ODPA is 1:0.925. (c) UV−vis absorption spectra and (d) FT-IR spectra of aliquots taken during direct synthesis of F393-InP:Zn MSCs from $In(Ac)_3$, ODPA, $(TMS)_3P$ and $Zn(SA)_2$ precursors at 300°C. The molar ratio of stearate to ODPA is 1:0.875.

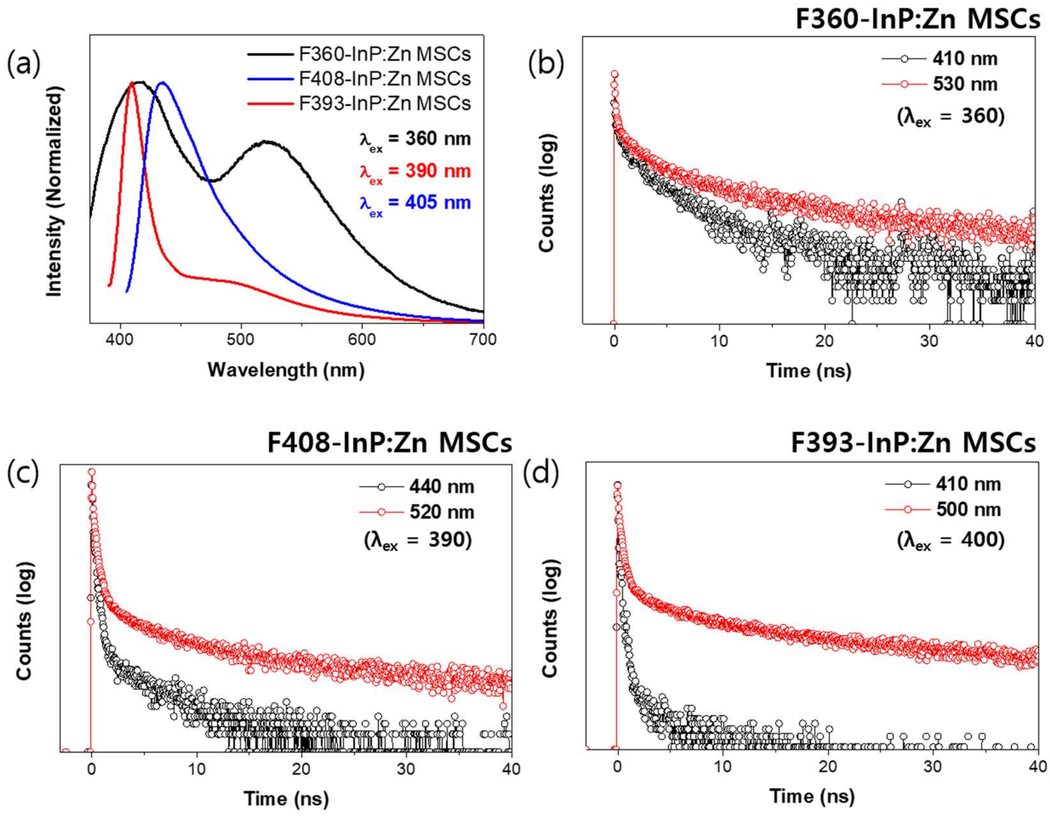

Supplementary Figure 26. (a) PL spectra of F360-InP:Zn MSCs, F408-InP:Zn MSCs, and F393-InP:Zn MSCs. Time-resolved PL decay profiles of (b) F360-InP:Zn MSCs, (c) F408-InP:Zn MSCs, and (d) F393-InP:Zn MSCs.

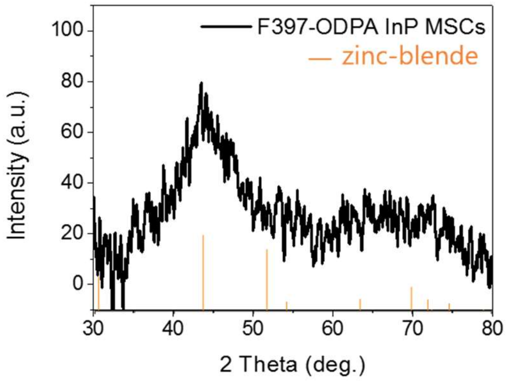

Supplementary Figure 27. XRD pattern of F397-ODPA InP MSCs.

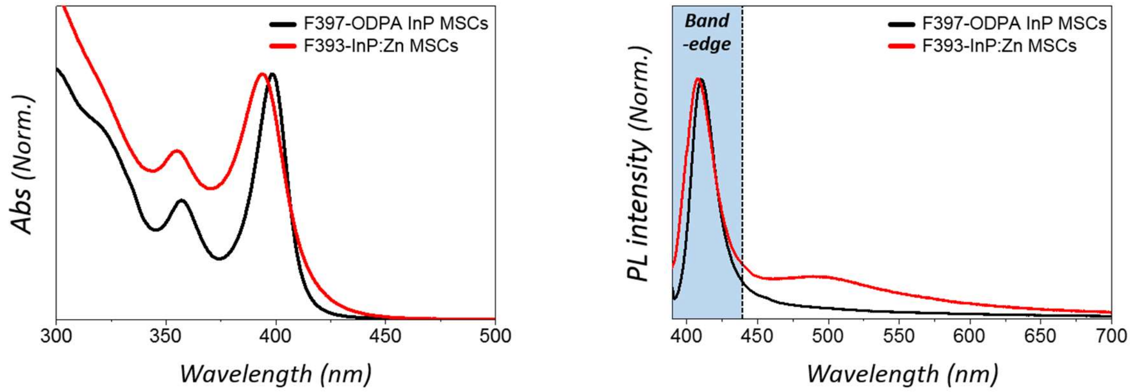

	F397-ODPA InP MSCs	F393-InP:Zn MSCs
FWHM (eV) *Band-edge*	**0.144**	**0.203**

	F397-ODPA InP MSCs	F393-InP:Zn MSCs
FWHM (eV) *Band-edge*	**0.148**	**0.194**
Q.Y. (%)	**1.84**	**2.0**
Q.Y. (%) *Band-edge*	**1.31**	**1.03**

Supplementary Figure 28. The steady-state absorption and PL spectra (top) of F397-ODPA InP MSCs and F393-InP:Zn MSCs with their absorptive and emissive properties (bottom). The band-edge emission band is extracted with a Gaussian peak fitting method.

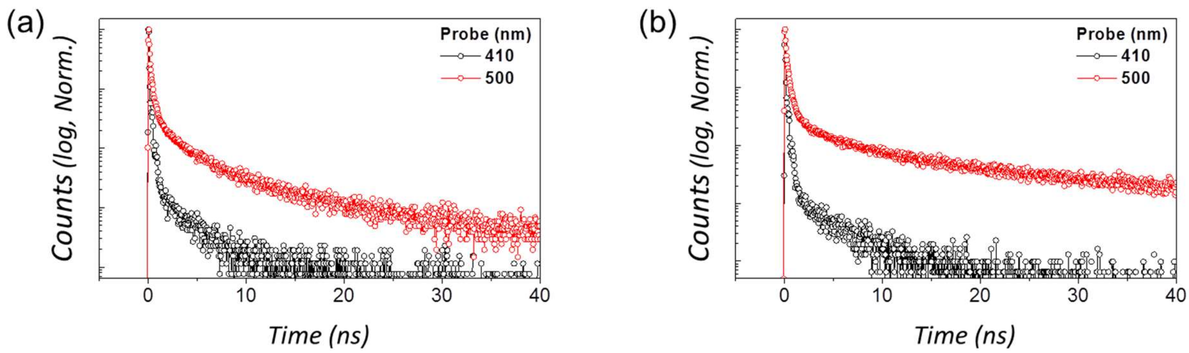

Supplementary Figure 29. Time-resolved PL decay profile of (a) F397-ODPA InP MSCs and (b) F393-InP:Zn MSCs.

Supplementary Reference

1 Gary, D. C.; Terban, M. W.; Billinge, S. J. L.; Cossairt, B. M., Two-Step Nucleation and Growth of InP Quantum Dots via Magic-Sized Cluster Intermediates. *Chemistry of Materials* **2015,** *27* (4), 1432.
